# Malvidin Protects against and Repairs Peptic Ulcers in Mice by Alleviating Oxidative Stress and Inflammation

**DOI:** 10.3390/nu13103312

**Published:** 2021-09-23

**Authors:** Felipe Leonardo Fagundes, Quélita Cristina Pereira, Melina Luzzi Zarricueta, Raquel de Cássia dos Santos

**Affiliations:** Laboratory of Pharmacology and Molecular Biology, Post Graduate Program in Health Sciences, Medical School, São Francisco University, Bragança Paulista, São Paulo 12916-900, Brazil; felipel_fagundes@hotmail.com (F.L.F.); quelitapereirapa@gmail.com (Q.C.P.); melina.luzzi@hotmail.com (M.L.Z.)

**Keywords:** malvidin, peptic ulcer, mice, anti-inflammatory activity

## Abstract

Peptic ulcer episodes cause damage to the stomach and intestine, with inflammatory cell infiltration and oxidative stress as the main players. In this study, we investigated the potential of anthocyanidin malvidin for preventive and curative peptic ulcer treatment. The anthocyanidin effects were examined in gastric ulcer mouse models induced by ethanol, non-steroidal anti-inflammatory drugs (NSAIDs), ischemia-reperfusion (IR), acetic acid and duodenal ulcer induced by polypharmacy. Expression levels of oxidative and inflammatory genes were measured to investigate the mechanism of anthocyanin activity. At a dose of 5 mg·kg^−1^, Malvidin prevented gastric ulcer induction by ethanol, NSAID and repaired the tissue after 6 days of IR. Moreover, the anthocyanidin accelerated the healing of acetic acid-induced ulcer, increased the gene expression of EGF and COX-1, and downregulated MMP-9. Anthocyanin treatment mitigated the effect of polypharmacy on inflammation and oxidative stress observed in the intestine. Additionally, the compound downregulated cytokine expression and TLR4 and upregulated HMOX-1 and IL-10, exhibiting protective activity in the mouse gut. Malvidin thus prevented gastric and duodenal ulcers due to prominent anti-inflammatory and antioxidative effects on the gastrointestinal tract that were related to gene expression modulation and an increase in endogenous defense mechanisms.

## 1. Introduction

Peptic ulcer is the most common gastrointestinal disorder typically observed in the stomach or small intestine. Its estimated prevalence is 10–15% globally, but this range might underestimate the prevalence of duodenal lesions. The main etiological factor is *Helicobacter pylori* infection, but other factors, such as treatment with non-steroidal anti-inflammatory drugs (NSAIDs), chronic stress, or alcohol consumption can trigger disease initiation [1]. Peptic ulcers are commonly treated with proton pump inhibitors (PPIs) or H2 blockers. The treatment limits chloride acid secretion, but it does not contribute to the healing phase. Moreover, continuous PPI use increases the risk of gastric adenocarcinoma and causes various severe side effects, including hepatotoxicity, headache, and in rare cases, Cushing syndrome [2]. Currently, PPIs are also associated with infection by *Clostridium difficile* and the development of pseudomembranous colitis [3]. Hence, the occurrence of severe adverse events limits the patient’s quality of life; specifically, it results in treatment suspension and recurrence. The use of NSAIDs combined with other drugs to treat a wide range of diseases such as rheumatoid arthritis and myocardial infarction has resulted in a practice known as polypharmacy, which is common in older patients. Physicians typically manage the upper gastrointestinal side effects of NSAIDs with PPIs, but novel studies indicate that anti-inflammatory drugs combined with PPI can exacerbate the lesions in the small intestine [4].

Anthocyanidins are polyphenolic plant pigments found in fruits and vegetables. They typically have two or three substituents, including a sugar and frequently an organic acid group. According to the Nurse’s Health Study II, the daily consumption of anthocyanidins is approximately 2–35 mg·kg^−1^ in women [5]. A single serving of berries can supply 100–200 mg of anthocyanidins, which are 5–10-fold more abundant than other flavanols [6]. Malvidin is an anthocyanidin present in grapes, wine, and black rice in the glycoside and aglycone form [7]. Different pharmacological effects of Malvidin have been reported in the literature, including the prevention of cardiovascular diseases, cancer, and diabetes, along with anti-inflammatory and antioxidant activities [8,9,10]. However, their effects on gastric or duodenal ulcers have not been examined. In this study, we investigated the potential of Malvidin for the prevention and treatment of peptic ulcers in mouse models and characterized their mechanisms of action.

## 2. Material and Methods

### 2.1. Chemical Compounds

Malvidin hydrochloride (>98% purity) was obtained from Cayman Chemical Company (Ann Harbor, MI, USA; PubChem ID 69512). Lansoprazole and omeprazole were purchased from Medley Sanofi (Campinas, SP, Brazil; PubChem ID 3883 and 4594, respectively). Tri Reagent, HTAB (PubChem ID 5974), Alcian Blue (PubChem ID 129628421), acetyl salicylic acid (PubChem ID 2244), indomethacin (PubChem ID 3715), sodium carbonate (PubChem ID 10340), DMSO (PubChem ID 679), Purpald (PubChem ID 2723946), xanthine oxidase from microorganism, orto-dianisidine hydrochloride (PubChem ID 12309823), sodium hydroxide (PubChem ID 14798), potassium hydroxide (PubChem ID), potassium dihydrogen phosphate (PubChem ID 516951), sodium orthovanadate (PubChem ID 61671), sodium pyrophosphate (PubChem ID 24403), sodium fluoride (PubChem ID 5235), Triton X-100 (PubChem ID 5590), and reduced glutathione (PubChem ID 124886) were obtained from Sigma Aldrich (Saint Louis, MO, USA). Xylazine hydrochloride (PubChem ID 68554) and ketamine hydrochloride (PubChem ID 15851) were obtained from Sespo Industry (Paulínia, SP, Brazil). Potassium periodate (PubChem ID 516896), absolute ethanol (PubChem ID 702), hydrogen peroxide (PubChem ID 784), and acetic acid (PubChem ID 176) were from Synth Chemistry (São Paulo, SP, Brazil). Methanol (PubChem ID 887) and hydrochloric acid (PubChem ID 313) were acquired from J.T Backer (Xalostoc, Mexico). Hypoxanthine (PubChem ID 135398638), nitrobluetetrazolium (PubChem ID 9281), and DTNB (PubChem ID 6254) were obtained from Alfa Aesar (Tewksbury, MA, USA). EDTA (PubChem ID 6049), Tris (PubChem ID 6503), protease inhibitor cocktail, bovine serum albumin, and catalase from bovine liver were obtained from Thermo Fischer Scientific (Amherst, MA, USA). Celecoxib (PubChem ID 2662) was acquired from Tocris Bioscience (Bristol, UK). PMSF (PubChem ID 4784) was from Amresco company (Solon, OH, USA), and sodium dodecyl sulfate (PubChem ID 3423265) was purchased from Merck (Darmstadt, Germany).

### 2.2. Animals

Animal studies were conducted following the ARRIVE GUIDELINES. Male Swiss mice (*Mus musculus*) were provided by the Multidisciplinary Center for Biological Investigation in the Area of Laboratory Animals (Unicamp, SP, Brazil) after approval by the Ethics Committee in Animal Use of São Francisco University (protocol number 001.12.2017). The animals were maintained in standard conditions (12 h light/dark cycle and room temperature of 22 ± 2 °C), fed with *Presence* diet and with free access to water. All the experiments were conducted according to international and Brazilian standards of animal welfare. The animals were randomly divided in groups for all experiments. The dose chosen for the positive control was based on the experiments in previous results obtained by our group an in the references in the literature [11].

### 2.3. Gastric Ulcer Induced by Absolute Ethanol

The mice were divided in four experimental groups including naïve, vehicle (saline solution NaCl 0.9%), positive control (Lansoprazole 30 mg·kg^−1^, a proton pump inhibitor that blocks gastric lesion), and Malvidin (5 mg·kg^−1^), based in previous experiments done by our group (*unpublished data*) *n* = 5. The drugs were administered by oral gavage one hour before giving absolute ethanol (0.2 mL/animal). Sixty minutes later, the animals were euthanized by administration of xylazine and ketamine (1:1 v,v) and the stomachs were collected for analyses of macroscopic lesion area using Av Soft Bio View Spectra (Paulínia, SP, Brazil) and oxidative and inflammatory parameters [12].

### 2.4. Gastric Ulcer Induced by Non-Steroidal Anti-Inflammatory Drug

The mice were divided into four different groups including vehicle (saline solution NaCl 0.9%), positive control (Lansoprazole 30 mg·kg^−1^), test group (Malvidin 5 mg·kg^−1^), or naïve (animals that did not receive any treatment) *n* = 5. Briefly, fasted animals were pre-treated with the control and the test compound by oral gavage 1 h prior to Indomethacin (50 mg·kg^−1^) administration. Six and a half hours later, the mice were euthanized with xylazine and ketamine overdose (1:1 v,v) and the stomachs were collected for macroscopic analyses of lesion area using Av Soft Bio View Spectra and to verify oxidative and inflammatory parameters [13,14,15].

### 2.5. Gastric Ulcer Induced by Ischemia-Reperfusion

The preventive model animals were divided into test (Malvidin 5 mg·kg^−1^) positive control (Lansoprazole 30 mg·kg^−1^), negative control (saline solution NaCl 0.9%) and sham (only operated, but not received any treatment) *n* = 3 and were submitted to the model of gastric injury induced by ischemia-reperfusion. Fasting mice were anesthetized with the combination of xylazine (8 mg·kg^−1^) and ketamine (80 mg·kg^−1^) and a laparotomy was made to access the left gastric artery. Then, a micro clamp was inserted, and the gastric circulation was interrupted for 30 min. Subsequently, the clap was removed, and reperfusion occurred for one hour. Then, the animals were euthanized with a combination of xylazine hydrochloride and ketamine (1:1 v,v) and the stomachs were collected to analyze macroscopic lesion area and verification of oxidative and inflammatory parameters [16,17,18]. In the curative model, the same surgical procedures were conducted, but the animals did not receive any pre-treatment, and 24 h after the surgery, they received oral administration of Malvidin (5 mg·kg^−1^), Lansoprazole (30 mg·kg^−1^), and Vehicle for 6 days (*n* = 5). Sham animals were included to validated the experiment. In the seventh day, the mice were euthanized, and the tissues were collected for macroscopic, inflammatory, and oxidative analyses. All the drugs used in these experiments were administered by oral gavage.

### 2.6. Gastric Ulcer Induced by Acetic Acid

The animals were divided into the naïve (did not receive any treatment), vehicle (saline solution NaCl 0.9%), Lansoprazole (30 mg·kg^−1^), and test (Malvidin 5 mg·kg^−1^) groups (*n* = 7) and were submitted to the model of gastric ulcer induced by acetic acid. Briefly, fasted mice were anesthetized with a combination of xylazine hydrochloride (8 mg·kg^−1^) and ketamine (80 mg·kg^−1^) and the abdomen were opened to expose the stomach. In the serosal surface, a 4.2 mm micro tube was positioned and 20 μL of acetic acid 80% (*v*/*v*) were placed in the tube for 35 s. Subsequently, the organ was cleaned with heat saline solution (NaCl 0.9%) and the abdomen was closed. Twenty-four hours after the surgery, the oral treatment was initiated for 14 days. On the fifteenth day, the animals were euthanized using xylazine and ketamine (1:1 v,v) and the ulcer region was collected to analyze the macroscopic lesion area, perform RT-PCR, and biochemical parameters [19,20,21].

### 2.7. Duodenal Ulcer Induced by Polypharmacy

Animals were divided into the naïve, test (Malvidin 5 mg·kg^−1^), and vehicle (saline solution) groups (*n* = 7) and were submitted to the model of duodenal ulcer induced by polypharmacy. The mice received omeprazole (20 mg·kg^−1^) daily and on the second day were administered with acetyl salicylic acid (10 mg·kg^−1^). On the fifth day, the animals received Celecoxib (10 mg·kg^−1^ two times a day). On the ninth day, the animals received the intervention (Malvidin 5 mg·kg^−1^). All drugs administered were by oral gavage. Five days after the beginning of treatment with the experimental compound, the mice were euthanized with a combination of xylazine and ketamine (1:1 v,v), and samples of the duodenum were collected to determine inflammatory and oxidative parameters [22,23]. 

### 2.8. Antioxidant and Inflammatory Parameters

Samples of stomach and duodenum were homogenized in the extraction buffer to determine the levels of catalase (CAT), reduced glutathione (GSH), and superoxide dismutase (SOD). The tissue homogenate was centrifuged at 14,000 rpm for 45 min and the supernatant was collected for enzymatic determination. Protein concentration was determined by biuret method using the Protal Kit (Laborclin, Minas Gerais, Brazil). For detailed protocols, consult the following work [24,25,26]. Myeloperoxidase was determined in 540 nm in the presence of hydrogen peroxide and o-dianisidine [27]. The results were expressed in units per gram of tissue similar to previously published works. Cytokine levels of tumor necrosis factor alpha (TNF-α), interleukin 1 beta (IL-1β), interleukin 6 (IL-6), and interleukin 10 (IL-10) were measured by enzyme-linked immunosorbent assay (ELISA) using mouse cytokines ELISA kits from R&D Systems according to the manufacturer’s protocol.

### 2.9. Quantitative qPCR Analyses

Stomach and duodenal strips, maintained in RNA later solution (Invitrogen, California, USA), were extracted using the Trizol Reagent. For RT-PCR, total RNA was reverse transcribed using the High-Capacity cDNA Reverse Transcription Kit (Applied Biosystems, Foster City, CA, USA). The synthesized cDNAs were amplified with Go Taq Q PCR Master Mix (Promega, Madison, WI, USA) and the reaction was performed using a 7300 Detection System. The relative amount of the target gene was calculated using the −2^ΔΔCT^ method with β-actin as a housekeeping gene. Primer pairs were designed based in the validated FASTA sequence using NCBI database and employing Primer3 software following the parameters: primer length 20–22 bases, melting temperature 60 °C, and amplicon size between 95 and 110 base-pair [28,29,30] (see in Table 1).

### 2.10. Statistical Analyses

The Lilliefors normality test was applied to verify the data normality in all data sets. Results were expressed as mean ± s.e.m and One Way Analyses of Variance (ANOVA) were applied to verify the difference between the means followed by the Dunnett’s test or Student T test. Statistical analyses were performed using Graph Pad Prism 8.0 (San Diego, CA, USA). A *p*-value less than 0.05 was considered significant.

## 3. Results

### 3.1. Malvidin Protected the Gastric Mucosa from Lesions Induced by Absolute Ethanol and Ameliorated the Inflammatory Parameters in the Stomach

**Figure 1:**
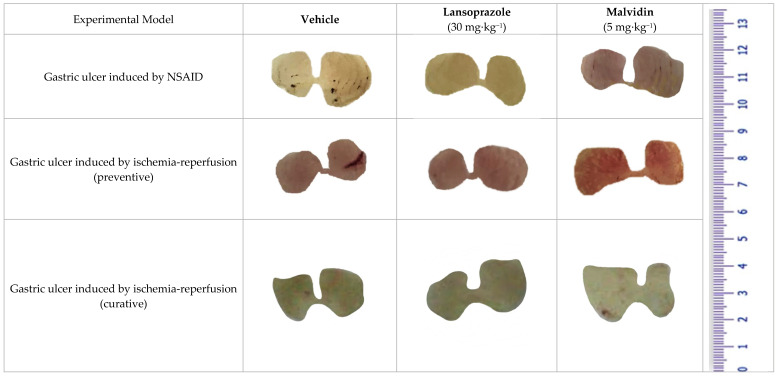
Macroscopic images of mice stomach treated with vehicle, lansoprazole (30 mg/kg) and malvidin (5 mg/kg) submitted to gastric ulcer induced by indomethacin (NSAID) and ischemia-reperfusion (both preventive and curative models).

To examine the potential gastroprotective effects of Malvidin, we used a mouse model of acute gastric lesions induced by absolute ethanol. We observed that the compound could inhibit the induction of lesions with a dose of 5 mg/kg (Figure 1). The potential anti-inflammatory activity of Malvidin in the gastric tissue was tested using the myeloperoxidase (MPO) assay. We observed that Malvidin reduced the MPO activity at the same dose that promoted gastroprotection. The positive control lansoprazole also promoted a significant reduction in MPO activity compared to the vehicle treatment (51.83%). To further examine the underlying mechanism involved in the gastroprotection promoted by Malvidin, we measured the activity of endogenous redox enzymes. We observed that anthocyanidin did not prevent the depletion of superoxide dismutase (SOD) and catalase compared with that of the vehicle treatment. Neither affected the reduced glutathione (GSH) level (see in Table 2).

### 3.2. Malvidin Exerts a Protective Effect in the Indomethacin-Induced Gastric Ulcer Model by Promoting a Reduction in Inflammatory and Oxidative Markers

To investigate the effect of Malvidin in the presence of NSAIDs, we performed an experiment in mice with indomethacin-induced gastric ulcer. In this model, a dose of 5 mg·kg^−1^ of the compound inhibited the formation of lesions when compared to the vehicle treatment groups (Figure 1). Similar results were obtained with lansoprazole using a dose of 30 mg·kg^−1^. Pre-treatment with Malvidin reduced the neutrophil infiltration measured as the MPO activity in stomach samples (35.84%). To investigate the involvement of oxidative markers in anthocyanidin-promoted gastroprotection, we measured the activity of antioxidant enzymes and levels of GSH. Malvidin did not elevated the activity of SOD and CAT, but increased the GSH levels (1.76-fold) compared with those in the vehicle-treated group. Lansoprazole did not significantly modulate the oxidative markers in the indomethacin-induced model compared with the vehicle-treated group (see Table 2). 

### 3.3. Malvidin Did Not Protect the Stomach from Macroscopic Alterations Induced by Ischaemia-Reperfusion, but Modulated Antioxidant Enzymes in Both Preventive and Curative Models

Pre-treatment with anthocyanidin Malvidin did not exert a protective role in the stomach of mice subjected to the ischemia–reperfusion protocol. The compound increased the macroscopic lesion area in the treated mice compared with that in the vehicle group (Figure 1). Only the control group did not show any lesions. Oral anthocyanidin administration did not prevent the neutrophil infiltration in the gastric tissue compared with that of the vehicle treatment. However, Malvidin affected the redox status, increasing the activity of SOD (4.14×) and CAT (2.87×) and elevating the levels of GSH (3.96×). The positive control lansoprazole did not substantially affect inflammatory and oxidative parameters compared with that of the vehicle treatment (see in Table 3). In the curative model, Malvidin did not reduce the macroscopic lesion area when compared with the vehicle treated group; however, it maintained the antioxidants enzymes elevated (see in Table 3). After 6 days of anthocyanidin treatment, we observed a decrease in the myeloperoxidase activity, showing a time dependent mechanism of Malvidin related to neutrophil infiltration.

### 3.4. Malvidin Accelerates Gastric Ulcer Healing in Mice with Acetic Acid-Induced Gastric Ulcer by Modulating the Expression Levels of EGF, COX-1, and MMP-9

**Figure 2:**
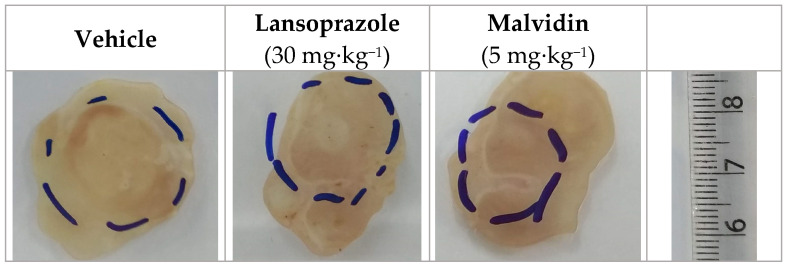
Macroscopic images of mice stomach submitted to gastric ulcer induced by acetic acid and treated for 14 days with vehicle, lansoprazole (30 mg/kg) and malvidin (5/mg/kg). The dashed line represents the ulcer size and localization.

**Figure 3:**
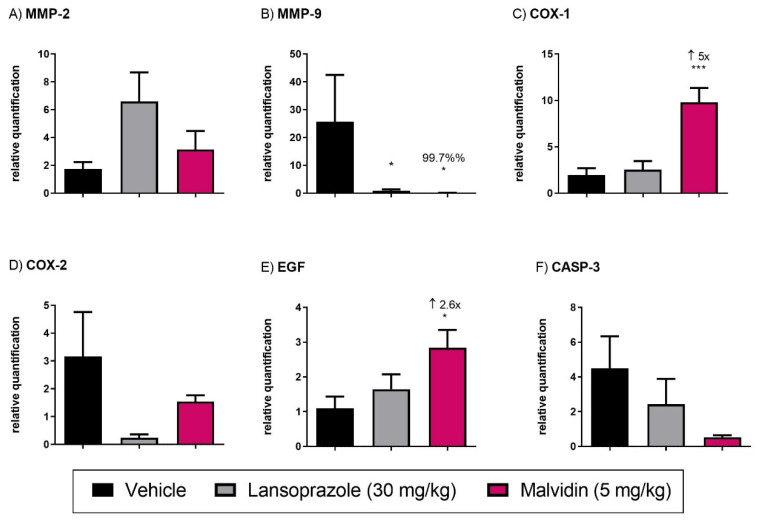
(**A**) MMP-2, matrix metalloproteinase (**B**) MMP-9, matrix metalloproteinase (**C**) COX-1 cyclooxygenase 1 (**D**) COX-2, cyclooxygenase 2 (**E**) EGF, epidermal growth factor and (**F**) CASP-3, caspase-3. The results show the relative gene expression of vehicle, lansoprazole (30 mg/kg) and malvidin (5 mg/kg) treated animals submitted to duodenal ulcer induced by polypharmacy. Data are presented as mean ± S.E.M. ANOVA followed by Dunnett′s test * *p* < 0.05 *** *p* < 0.001 represents difference in relation to control group treated with vehicle.

Daily treatment for 14 days with anthocyanidin promoted the reduction in the lesion area, as measured with a digital pachymeter (see Figure 2). Malvidin was also found to reduce the ulcer scar to 36.24% compared with the vehicle-treated group which received saline for the same period. The positive control (lansoprazole, 30 mg·kg^−1^) also reduced the scar area compared with the vehicle group. Treatment with anthocyanidins altered the gene expression of relevant targets involved in gastric healing. Malvidin elevated the expression of the EGF gene (2.6-fold), encoding epidermal growth factor, and COX-1 (5-fold), encoding cyclooxygenase-1, as compared to those in the vehicle-treated mice. However, Malvidin did not change the expression levels of matrix metalloproteinase 2 but reduced the relative expression of MMP-9 (99.7%) (see Figure 3). 

### 3.5. Malvidin Improves Antioxidant Defense in the Gastric Mucosa of Mice with Acetic acid-Induced Gastric Ulcer

Over 14 days of anthocyanidin treatment, Malvidin elevated the SOD activity in the gastric mucosa compared with that in the vehicle-treated group (2.68-fold). Malvidin did not promote alterations in the glutathione levels or CAT activity. The positive control lansoprazole did not modulate any of the tested antioxidants, exhibiting no effect on oxidative defense. The elevated redox status due to anthocyanidin administration was superior to that observed in the sham animals, indicating a substantial improvement (see Table 4). 

### 3.6. Anthocyanidins Ameliorate the Inflammatory Status and Oxidative Parameters in Mice with Polypharmacy-Induced Duodenal Ulcer

Anthocyanidin treatment for 5 days reduced the MPO activity in the duodenum of mice subjected to polypharmacy compared with that in vehicle-treated mice (61.32%). Moreover, Malvidin elevated the CAT activity in intestine samples (1.86-fold) but did not change the other oxidative parameters (i.e., SOD and GSH). Additionally, we investigated the efficacy of anthocyanidin in inhibiting cytokine production in the mouse model of polypharmacy. NSAID and PPI treatment elevated the release of proinflammatory mediators from immune cells, but treatment with 5 mg·kg^−1^ of Malvidin decreased the tissue content of two major inflammatory mediators, tumor necrosis factor α (TNF-α) and interleukin 6 (IL-6) (60.56% and 53.27%, respectively). Malvidin did not affect interleukin 1β (IL-1β) and interleukin 10 (IL-10) expression (see Table 5).

### 3.7. Malvidin Does Not Prevent Expression Profile Changes in Tight Junction Factors in the Duodenum of Mice with Polypharmacy-Induced Duodenal Ulcer

**Figure 4:**
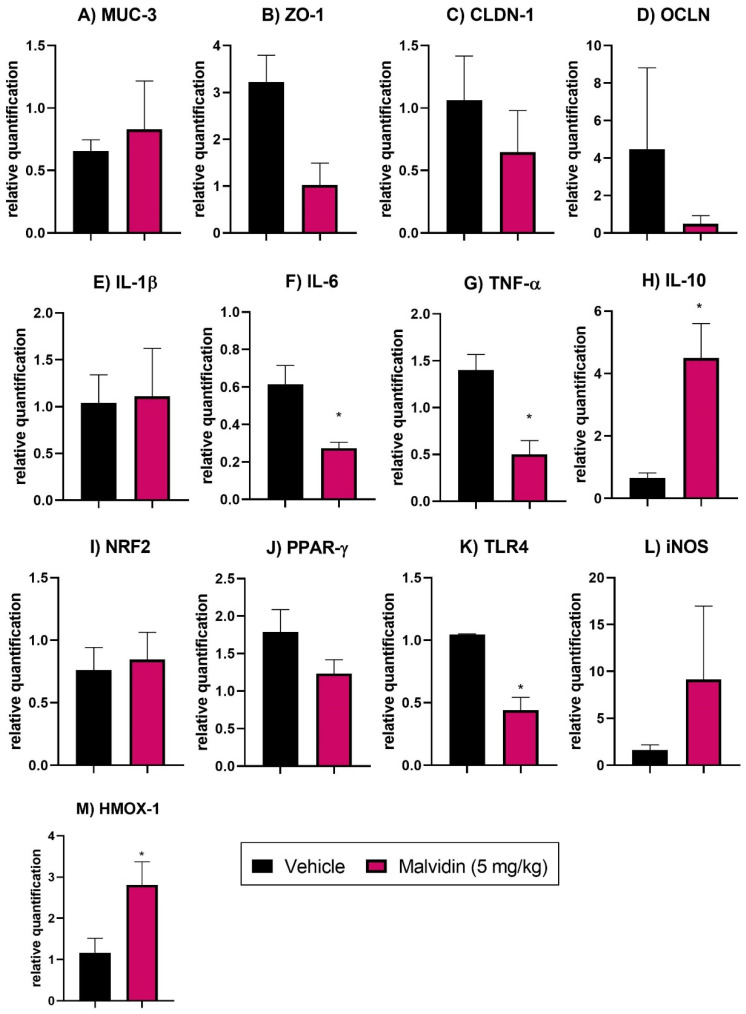
(**A**) MUC-3, mucin 3 (**B**) ZO-1, zonula occludens 1 (**C**) CLDN-1,claudin 1 (**D**) OCLN, occludin (**E**) IL-1β, interleukin 1 beta (**F**) IL-6, interleukin 6 (**G**) TNF-α, tumor necrosis factor alfa (**H**) IL-10, interleukin 10 (**I**) NRF2, nuclear factor erythroid 2-related factor 2 (**J**) PPAR-γ, peroxisome proliferator-activated receptor gamma (**K**) TLR4, toll like receptor 4 (**L**) iNOS, inducible nitric oxide synthase (**M**) HMOX-1, heme oxygenase 1. The results show the relative gene expression of vehicle or malvidin (5 mg/kg) treated animals submitted to duodenal ulcer induced by polypharmacy. Data are presented as mean ± S.E.M. Parametric Student T Test * *p* < 0.05 represents difference in relation to control group treated with vehicle.

RT-PCR of duodenum samples for tight junction genes revealed substantial expression level alterations in ZO-1, encoding zonula occludens-1, CLDN-1, encoding claudin-1, and OCLN, encoding occludin, in the vehicle-treated group. Treatment with anthocyanidin malvidin, in turn, did not elevate any tight junction proteins (see Figure 4). 

### 3.8. Malvidin Downregulates Genes Related to Inflammation, Oxidative Stress, and Immune System Activation in the Polypharmacy-Induced Duodenal Ulcer Model

RT-PCR analyses of small intestine samples revealed that vehicle-treated animals exhibited high expression levels of the proinflammatory cytokine genes such as IL-6, and TNF-α. Moreover, anti-inflammatory cytokine genes, such as IL-10, were downregulated. Malvidin reduced the relative expression of IL-6 (55.74%) and TNF-α (64.29%) in the duodenum and elevated the expression of IL-10 (6.92-fold), indicating a potent anti-inflammatory mechanism. Moreover, it elevated HMOX-1 (heme oxygenase 1) and reduced TLR4 (toll-like receptor 4) expression in the small intestine (see Figure 4).

## 4. Discussion

The exposure of gastric mucosa to absolute ethanol is a classical experimental model of gastric ulcer used to determine the efficacy of lead compounds in the preventive treatment of peptic ulcer. The presence of alcohol causes a variety of cellular and oxidative damage, increases lipid peroxidation and production of leukotrienes, reduces glutathione content and sulfhydryl groups, and promotes neutrophil infiltration [31,32]. Anthocyanidin treatment inhibited the formation of macroscopic lesions in the gastric mucosa and reduced the inflammatory biomarker MPO. The myeloperoxidase activity represents one of the main indicators of gastric injuries [33], but it cannot elevate the activity of the main antioxidant enzymes catalase and superoxide dismutase. We believe that this dose was not sufficient to promote alterations in SOD activity based in the previous publish works, in which Malvidin was used in a dose 20 times higher [34]. Moreover, Malvidin did not elevate the levels of GSH, mainly because the compound could not activate the enzymes responsible for Glutathione synthesis in the cytoplasm of gastric cells.

We evaluated the response of the gastric mucosa to an anthocyanidin after induction with NSAID indomethacin. This gastric ulcer model is associated with excessive production of reactive oxygen species (ROS) during the ulceration process, leading to the oxidation of proteins and depletion of antioxidant defenses [35]. The production of ROS leads to lipid peroxidation, oxidation of intracellular proteins and nucleic acids, and the disruption of normal cell function [36]. Moreover, the presence of indomethacin elevates the release of proinflammatory mediators and promotes neutrophil infiltration [37]. In this model, Malvidin reduced the macroscopic lesion area, neutrophil influx, and increased the antioxidant defenses by blocking GSH depletion and elevating SOD activity. Published data showed similar effects of Malvidin found in the açai fruit [38].

To test the hypothesis that anthocyanidins are potent antioxidants in the gastrointestinal tract, we performed gastric ischemia–reperfusion in mice. Recent evidence showed that microvascular alterations and the generation of free radicals are the initiating events of mucosal injury induced by ischemia [39]. The ischemia process also induces cell death and mucosal injury that depends on the acute inflammatory response and oxidative damage [40]. In our study, Malvidin neither prevented the macroscopic lesions nor reversed the neutrophil infiltration in the preventive model. However, the compound increased the levels of selected antioxidant factors, confirming their protective role in the oxidative process. The scavenging activities of these compounds is not sufficient to prevent the inflammatory and oxidative consequences of ischemia–reperfusion in the gastric tissue [41]. At the end of the ischemic period, there is a return of both blood flow and oxygen level. However, the lower concentration of antioxidants in ischemic cells triggers an increase in the generation of local ROS, which results in oxidative stress, leading to endothelial dysfunction, DNA damage, and inflammatory response in the cascade at the site that could induce cell death [39,42]. As reported in the literature, the isolates of polyphenolic compounds cannot be used to treat oxidative disorders caused by inflammatory components. In the curative model, Malvidin exhibited the same behavior related to macroscopic lesion area; otherwise, the anthocyanidin treatment for six days reduced the neutrophil infiltrate measured by myeloperoxidase activity, showing a time dependent fashion in the gastric mucosa exposed to ischemia and reperfusion. These can be explained by the microvascular actions of anthocyanidins. In the absence of blood flow, the molecule cannot inhibit the leukocyte recruitment, which is related to the increase in lesion ratio and that it is regulated by inflammatory signals provided by immune cells in the lesion area. When the normal flow is restored, as observed in the curative model, there is a reduction in granulocyte presence, related to Malvidin treatment and probably a better bioavailability of the compound.

Our next experiment tested the healing potential of anthocyanidin in the gastric ulcer induced by acetic acid. This model closely mimics human ulcers and is useful for searching novel anti-ulcerogenic compounds [43]. The tested anthocyanidin increased tissue recovery after acetic acid exposure and downregulated genes related to matrix remodeling and inflammation and upregulated genes associated with cell growth. The chronic ulcers caused the elevation of matrix metallopeptidase-9, which is responsible for extracellular matrix degradation, indicating inflammation and ineffective healing [44,45]. Malvidin downregulated MMP-9 expression showing that anthocyanidin can control matrix degradation, contributing to ulcer healing. Other events observed during these processes are related to inflammation, which plays an important role in tissue repair. Anthocyanidin upregulated COX-1 expression, indicating selective activity affecting the cyclooxygenase enzymes that are crucial in the production of lipid mediators responsible for gastric maintenance and mucosal integrity, such as prostaglandin E2 [46,47]. Furthermore, this treatment also upregulated the EGF gene which promotes cell proliferation. As indicated in the literature, the EGF protein is critical for the proliferation and differentiation of gastric cells, as well as the regulation of glandular morphogenesis in the stomach [48]. During the healing process, there are alterations in the redox status of the gastric mucosa that are a consequence of immune cell infiltration and the release of ROS [49,50]. Malvidin could substantially restore the antioxidant defenses in gastric tissue by elevating SOD activity and GSH levels above baseline, indicating that these factors are fundamental in tissue repair. 

The last model in our study was the duodenal ulcer induced by polypharmacy, which has physiopathological characteristics that differ from those observed in gastric ulcer. The presence of NSAIDs in the small intestine can cause alterations in the gut permeability and result in the production of specific lesions that are diagnosed through endoscopy [51,52]. The combination of selective NSAIDs with a PPI, which is used in clinics to prevent lesions in the stomach, is ineffective in preventing small intestinal injury, as demonstrated in clinical studies [53]. The combination of PPI, NSAIDs, and an anti-platelet aggregator, commonly used in patients with chronic pain and myocardial infarction, has been implicated in the pathophysiology of duodenal ulcer induced by polypharmacy [23]. NSAIDs disrupt the mucus barrier and change the commensal microbiota that protects the host from pathogenic organisms [54]. During the enteropathy caused by NSAIDs, we observed alterations in the expression of critical tight junction proteins, ZO-1, CLDN-1, and OCLN. These proteins regulate intestinal permeability and control the transit of substances, nutrients, bacteria, and toxins across the intestinal epithelia [55,56]. The anthocyanin treatment did not reverse the increased intestinal permeability observed in the vehicle group. The activation of TLR4 by bacterial lipopolysaccharide present in the gut lamina propria induces intracellular responses, such as the increase of the nuclear factor kappa B and induction of the transcription of inflammatory cytokines, including IL-6, IL-1β, and TNF-α [57]. The presence of Malvidin downregulated TLR4, demonstrating a protective role in the small intestine that is possibly related to a reduction in microbial sensing. Animals treated with the compound had lower expression levels of the proinflammatory cytokine TNF-α and IL-6, which are mainly secreted by macrophages and associated with a pro-inflammatory state. The presence of inflammatory cells also results in oxidative damage in the gut, which reduces antioxidant defenses. Nuclear erythroid factor 2-related factor 2 (NRF2) is a transcription factor associated with antioxidant enzymes, which plays a master role in redox homeostasis in the cells [58]. We observed that the administration of Malvidin did not change NRF2 expression levels, but increased CAT activity in the compound-treated groups, indicating that the NRF2 transcription factor is not a target of anthocyanin action. The elevated CAT activity is independent from NFR2 regulation and is not fully understood in the literature [59]. The administration of anthocyanidin also increased HMOX-1 expression, a key enzyme related to endogenous carbon monoxide release, which contributes to gastroprotection and is associated with the anthocyanin effects in the polypharmacy model [60,61].

## 5. Conclusions

Our study provided evidence for the pharmacological effects of Malvidin on peptic ulcer disease for the first time. This activity is associated with the reduction in inflammatory biomarkers, such as MPO, and an increase in endogenous antioxidant defenses, such as CAT, SOD, and GSH. Furthermore, it displayed preventive and healing capacity in the mouse models of gastrointestinal disorders. Treatment with absolute ethanol, indomethacin, and ischemia–reperfusion caused cellular and oxidative damage that Malvidin prevented by modulating redox system enzymes and inflammatory markers. In the acetic acid model, the compounds stimulated EGF and COX-1 gene expression and reduced MMP-9 expression, exhibiting target selectivity during the healing process. Additionally, anthocyanidins are novel therapeutic resources used to treat enteropathy induced by polypharmacy. Treatment with Malvidin improved the redox status in the small intestine and reduced the expression of proinflammatory genes, which is critical for maintaining the intestinal barrier and protecting the gut from microbial products. Based in these results and the general consumption of anthocyanidin by population, route of administration used in this study and the presence in fruits and foods, we recognize Malvidin as a promising molecule that can be used in the management of peptic ulcers by humans. Meanwhile, other studies are necessary to corroborate these findings. 

## Figures and Tables

**Table 1:** Genes selected to characterize the mechanism of action of malvidin in gastric ulcer induced by acetic acid and duodenal ulcer induced by polypharmacy in mice.

Gene	Sequence 5′-3′	GenBank Number
*MMP-2*	5′-GGACAGTGACACCACGTGAC-3′	17,390
	5′-TGACACAGCCTTCTCCTCCT-3′	
*MMP-9*	5′-CGTCGTGATCCCCACTTACT-3′	17,395
	5′-AACACACAGGGTTTGCCTTC-3′	
*CASP-3*	5′-GGCCGTGTTTCTGTTTTGTT-3′	12,367
	5′-TTGAGGTAGCTGCATGTGG-3′	
*COX-1*	5′-AGGGTGTCTGTGTCCGCTTT-3′	19,224
	5′-GTTGGGGACTGGAGTCTTGC-3′	
*COX-2*	5′-CCCCAAAGATAGCATCTGGA-3′	19,225
	5′-TGCAGAATTGAAAGCCCTCT-3′	
*EGF*	5′-AGGCATCAAGCACGGTAGGT-3′	13,645
	5′-AGCAAGCACACCCCGTAAGT-3′	
*iNOS*	5′-TGGTGGTGACAAGCACATTT-3′	18,126
	5′-AAGGCCAAACACAGCATACC-3′	
*B-actin*	5′-ACGAGGCCCAGAGCAAGAG-3′	11,461
	5′-GGTGTGGTGCCAGATCTTCTC’-3′	
*ZO-1*	5′-CTGTGGGTTCCGTTTTGAGT-3′	21,872
	5′-CAGAAGGCCAAAGACTCCAG-3′	
*CLND*	5′-AAAATCCCTGACGGGGTATC-3′	12,737
	5′-GGCGTTTCTGGATGTTGTCT-3′	
*OCLD*	5′-CTCACGGAAACCAGAGAAGC-3′	18,260
	5′-GCATTTCTGGTGGACAAGGT-3′	
*IL-1β*	5′-CCCAAGCAATACCCAAAGAA-3′	16,176
	5′-TACCAGTTGGGGAACTCTGC-3′	
*TNF-α*	5′-TAGCCAGGAGGGAGAACAGA-3′	21,926
	5′-TTTCTGGAGGGAGATGTGG-3′	
*IL-6*	5′-TCTCTGGGAAATCGTGGAA-3′	16,193
	5′-TTCTGCAAGTGCATCATCG-3′	
*IL-10*	5′-AAAAGGTGCCACCCTGAAGA-3′	16,153
	5′-GATGTGGTGGGACCAACCTT-3′	
*NRF2*	5′-CCCAGGGTTTGAAAAGTGAA-3′	18,024
	5′-GCTGGAAAGTGAAGGCAGTC-3′	
*HMOX-1*	5′-CGATCTCAAGCAAGCCCTAC-3′	15,368
	5′-TTGGTGAGTTCCTCCTTGCT-3′	
*TLR4*	5′-AGAAAATGCCAGGATGATGC-3′	21,898
	5′-AGGGATTCAAGCTTCCTGGT-3′	
*PPAR-γ*	5′-CCCTGGTGTCCCAACTCTTA-3′	19,016
	5′-GTGCAACAGAAGAGCCATCA-3′	

Target genes, primer sequence and GenBank number used in the RT-PCR analyses.

**Table 2:** The effect of treatment with malvidin in different models of gastric ulcer prevention in mice.

Experimental Model	Treatment (p.o.)	Dose (mg/kg)	Ulcerative Lesion (mm^2^)	Gastric Lesion Inhibition (%)	MPO (Unit of MPO/g)	GSH (nmol/g)	CAT (Unit of CAT/g)	SOD (Unit of SOD/g)
Gastric ulcer induced by absolute ethanol	Vehicle	-	243.0 ± 68.0	-	29.3 ± 4.3	827.3 ± 85.1	38.3 ± 6.3	16.6 ± 3.0
	Lansoprazole	30	50.7 ± 25.8	79.1 *	16.2 ± 1.5 *	962.2 ± 153.2 *	61.1 ± 6.1 *	14.9 ± 1.6
	Malvidin	5	25.3 ± 8.8	89.6 **	14.1 ± 1.0 *	627.5 ± 77.7	48.3 ± 4.2	10.2 ± 1.7
	Naive	-	-	-	16.8 ± 1.6 *	979.5 ± 79.1 **	87.9 ± 5.0 **	19.3 ± 1.9 *
Gastric ulcer induced by NSAID	Vehicle	-	27.1 ± 4.8	-	20.7 ± 2.0	99.2 ± 10.4	36.8 ± 4.5	10.3 ± 1.2
	Lansoprazole	30	1.7 ± 0.6	93.7 ****	15.2 ± 0.9 *	122.2 ± 12.3	44.3 ±5.7	13.3 ± 2.7
	Malvidin	5	9.8 ±3.3	63.8 **	13.3 ± 1.0 *	174.6 ± 5.8 **	50.8 ±2.2	16.2 ± 1.6
	Naive		-	-	10.4 ± 0.7 **	142.0 ± 13.1 *	56.6 ± 6.9 *	13.5 ± 1.1

The results show the effect of vehicle, lansoprazole (30 mg/kg) and malvidin (5 mg/kg) in the ulcerative lesion, activity of mieloperoxidase (MPO), catalase (CAT), superoxide dismutase (SOD) and in the levels of reduced glutathione (GSH) in gastric tissue from animals submitted to gastric ulcer induced by absolute ethanol and non-steroidal anti-inflammatory drug. Data are presented as mean ± S.E.M. ANOVA followed by Dunnett’s test. * *p* < 0.05, ** *p* < 0.01 and **** *p* < 0.0001 represents difference in relation to control group treated with vehicle.

**Table 3:** Comparative analysis of malvidin activity in ischemia-reperfusion induced ulcers.

Experimental Model Ischemia/Reperfusion	Treatment (p.o.)	Dose (mg/kg)	Ulcerative Lesion (mm^2^)	Gastric Lesion Inhibition (%)	MPO (Unit of MPO/g)	GSH (nmol/g)	CAT (Unit of CAT/g)	SOD (Unit of SOD/g)
Preventive treatment	Vehicle	-	30.3 ± 6.2	-	16.3 ± 1.4	58.4 ± 7.5	18.2 ± 3.3	3.4 ± 0.2
	Lansoprazole	30	2.4 ± 1.4	92.1 *	13.4 ± 1.6	32.4 ± 7.2	7.5 ± 0.4	2.5 ± 0.6
	Malvidin	5	633.6 ± 77.9	-	15.8 ± 1.2	231.3 ± 87.0 *	52.3 ± 12.4 ***	13.8 ± 3.1 ***
	Naive	-	-	-	12.2 ± 0.2	231.8 ± 73.1 *	29.8 ± 7.2 ***	14.5 ± 2.8 ***
6 days of post-ischemia-reperfusion treatment	Vehicle	-	5.93 ± 0.025	-	29.87 ± 6.36	48.05 ± 20.55	53.10 ± 6.03	11.64 ± 0.69
	Lansoprazole	30	0.38 ± 0.12	99.94	17.08 ± 2.65 *	59.69 ± 3.73	55.25 ± 7.97	9.99 ± 0.32
	Malvidin	5	19.23 ± 4.10	-	15.63 ± 0.76 *	95.65 ± 12.58 *	109.7 ± 12.96 *	14.02 ± 1.04
	Sham	-	-	-	11.95 ± 1.17 *	129.3 ± 0.00 *	63.71 ± 12.93	15.16 ± 1.16 *

The results show the effect of vehicle, lansoprazole (30 mg/kg) and malvidin (5 mg/kg) in the ulcerative lesion, activity of mieloperoxidase (MPO), catalase (CAT), superoxide dismutase (GSH) and in the levels of reduced glutathione (GSH) in gastric tissue from animals submitted to gastric ulcer induced ischemia-reperfusion. Data are presented as mean ± S.E.M. ANOVA followed by Dunnett′s test. * *p* < 0.05 and *** *p* < 0.001 represents difference in relation to control group treated with vehicle.

**Table 4:** Effect of Malvidin treatment for 14 days after acetic acid-induced gastric ulcer.

Experimental Model	Treatment (p.o.)	Dose (mg/kg)	Ulcerative Lesion (mm^2^)	Gastric Lesion Reduction (%)	GSH (nmol/g)	CAT (Unit of CAT/g)	SOD (Unit of SOD/g)
Acetic acid-induced gastric ulcer	Vehicle	-	7.1 ± 0.6	-	53.8 ± 6.7	26.7 ± 3.8	6.1 ± 0.6
	Lansoprazole	30	4.8 ± 0.5	32.4 *	72.05 ± 13.2	59.7 ± 18.2	7.2 ± 1.6
	Malvidin	5	4.9 ± 0.4	31.0 *	70.4 ± 17.4	35.5 ± 4.7	11.8 ± 1.5 *
	Sham	-	-	-	67.7 ± 14.8	33.2 ± 2.9	8.4 ± 1.8

The results show the effect of vehicle, lansoprazole (30 mg/kg) and malvidin (5 mg/kg) in the ulcerative lesion, activity of mieloperoxidase (MPO), catalase (CAT), superoxide dismutase (SOD) and in the levels of reduced glutathione (GSH) in gastric tissue from animals submitted to gastric ulcer induced by acetic acid. Data are presented as mean ± S.E.M. ANOVA followed by Dunnett′s test. * *p* < 0.05 represents difference in relation to control group treated with vehicle.

**Table 5:** Effect of malvidin treatment in the polypharmacy-induced duodenal ulcer model.

Experimental Model	Treatment (p.o.)	Dose (mg/kg)	MPO (Unit of MPO/g)	GSH (nmol/g)	CAT (Unit of CAT/g)	SOD (Unit of SOD/g)	IL-10 (pg/mL)	IL-6 (pg/mL)	IL-1β (pg/mL)	TNF-α (pg/mL)
Polypharmacy-induced duodenal ulcer	Vehicle	-	95.3 ± 19.4	734.9 ± 74.2	45.6 ± 6.0	12.3 ± 1.4	1791.0 ± 350.0	495.8 ± 167.7	6377.0 ± 1128.0	1433.0 ± 447.8
	Malvidin	5	36.9 ± 8.0 **	765.0 ± 66.4	84.8 ± 6.6 **	9.7 ± 2.0	1801.0 ± 326.1	193.2 ± 40.8 *	6056.0 ± 861.2	565.3 ± 91.1 *
	Naïve	-	27.3 ± 9.4 **	910.7 ± 50.6	84.0 ± 4.1 **	16.7 ± 0.5	1799.0 ± 494.7	223.4 ± 33.4	5595.0 ± 674.0	649.6 ± 112.6

The results show the effect of vehicle and malvidin (5 mg/kg) in the activity of mieloperoxidase (MPO), catalase (CAT), superoxide dismutase (SOD), levels of reduced glutathione (GSH) and cytokines concentration in the small intestine from animals submitted to duodenal ulcer induced by polypharmacy. Data are presented as mean ± S.E.M. ANOVA followed by Dunnett′s test. * *p* < 0.05 and ** *p* < 0.01 represents difference in relation to control group treated with vehicle.

## Data Availability

The data presented in this study are available on request from the corresponding author.
